# SP1 Gene Methylation in Head and Neck Squamous Cell Cancer in HPV-Negative Patients

**DOI:** 10.3390/genes15030281

**Published:** 2024-02-23

**Authors:** Enar Jumaniyazova, Anna Aghajanyan, Sergey Kurevlev, Leyla Tskhovrebova, Andrey Makarov, Konstantin Gordon, Anastasiya Lokhonina, Timur Fatkhudinov

**Affiliations:** 1 Institute of Medicine, Peoples’ Friendship University of Russia (RUDN University), 6 Miklukho-Maklaya Street, 117198 Moscow, Russia; 2 Histology Department, Pirogov Russian National Research Medical University, Ministry of Healthcare of the Russian Federation, 117997 Moscow, Russia; 3 A. Tsyb Medical Radiological Research Center, Branch of the National Medical Research Radiological Center of the Ministry of Health of the Russian Federation (A. Tsyb MRRC), 4, Korolev Street, 249036 Obninsk, Russia; 4 Avtsyn Research Institute of Human Morphology of Petrovsky National Research Centre of Surgery, 3 Tsyurupy Street, 117418 Moscow, Russia

**Keywords:** methylation of DNA, head and neck squamous cell cancer, epigenetic changes, *Sp1* gene

## Abstract

There is still much to learn about the epigenetic mechanisms controlling gene expression during carcinogenesis. When researching aberrant DNA methylation, active proliferative tumor cells from head and neck squamous cell cancer (HNSCC) can be used as a model. The aim of the study was to investigate the methylation status of *CDKN1*, *CDKN2A*, *MYC*, *Smad3*, *SP1*, and *UBC* genes in tumor tissue (control-normal tissue) in 50 patients (37 men and 13 women) with HPV-negative HNSCC. Methods: Bisulfite conversion methods and methyl-sensitive analysis of high-resolution melting curves were used to quantify the methylation of genes. In all patients and across various subgroups (tongue carcinoma, laryngeal and other types of carcinomas T2, T3, T4 status; age before and after 50 years; smoking and non-smoking), there are consistent differences in the methylation levels in the *SP1* gene in tumor DNA compared to normal. Results: The methylation of the *SP1* gene in tumor DNA suppresses its expression, hinders HNSCC cell proliferation regulation, and could be a molecular indicator of malignant cell growth. The study of DNA methylation of various genes involved in carcinogenesis is promising because hypermethylated promoters can serve as potential biomarkers of disease.

## 1. Introduction

Head and neck squamous cell cancer (HNSCC) is one of the ten most common malignant neoplasms [1]. Currently, active research is underway to discover and characterize new molecular genetic signatures of head and neck squamous cell carcinoma. Based on some of these signatures, the WHO HNSCC classification was recently improved in 2022. Thus, it has been shown that the nature of genetic changes is often associated with etiological factors that serve as a “trigger” for the development of oncopathology, in the development of HNSCC among the leading factors that increase the risk of developing the disease, infection with human papillomavirus, smoking, alcohol abuse. It is known that, unlike their HPV-positive counterparts, HPV-negative tumors are characterized by high mutational load and chromosomal aberrations with different copy number alteration (CNA) profiles [2]. HPV-positive HNSCC has a higher frequency of aberrant DNA methylation compared to HPV-negative HNSCC. Various studies have shown that various genes are frequently hypermethylated in HPV-positive HNSCC. Thus, HPV infection status affects not only the genetic alterations but also the prognosis of patients with HNSCC [3]. Compared to the DNA methylation profiles of HPV-positive HNSCC, fewer studies have been conducted to investigate the DNA methylation profiles of HPV-negative HNSCC, although it is this group of patients that needs the isolation of diagnostic and prognostic biomarkers due to their unfavorable prognosis [4].

The Cancer Genome Atlas characterized the molecular genetic landscape of 279 primary HNSCC of different anatomical localization, with the majority of patients (over 60%) having a long smoking history and being HPV-negative, only 13% were identified as HPV-positive (>60%) [5]. For our study, we decided to select a group of HPV-negative patients with HNSCC of different anatomical localization with different smoking status and to investigate them for the presence of epigenetic changes in 6 genes. The sample of patients is small and is characterized by anatomical heterogeneity, which may also influence the pattern of gene methylation.

HNSCC carcinogenesis is a multiserial process under the control of the genetic machinery of cancer cells. In addition to the well-studied genetic alterations that contribute to oncogenesis, the important role of epigenetic abnormalities is now also recognized [6].

A number of studies have demonstrated that carcinogenesis is accompanied by changes in cell DNA methylation. DNA methylation is an important tool for epigenetic regulation of gene expression in physiological conditions and in pathology, in particular, in cancer. It is characterized by global hypomethylation of the genome with focal hypermethylation of numerous 5′-cytosine-phosphate-guanine-3′-islands (CpG), often covering gene promoters and the first exons of genes involved in cell cycle regulation, which causes genome instability [7,8]. Most often, methylation occurs within genomic regions with a higher frequency of CpG nucleotides, which are predominantly localized in promoter regions. As a result of this promoter change, the affinity of transcription factors for target genes changes, as well as the mobilization of other proteins, such as methyl-binding domain proteins and chromatin remodelers [9]. In cases where DNA methylation is concentrated in the promoter region, it usually results in gene silencing. In contrast, when DNA methylation affects the core region of a gene, the result is usually an increase in gene expression [10]. To date, an increasing number of researchers tend to believe that malignant transformation of cells is preceded by a “breakdown” of the cell genome, consisting in the suppression of a tumor suppressor gene and/or activation of a pro-oncogene, to which epigenetic changes may lead. In this paper, we decided to study the methylation of the following genes: *CDKN1*, *CDKN2A*, *MYC*, *Smad3*, *SP1*, and *UBC*. These genes were selected based on a literature review. Characteristics of the analyzed genes are presented in Table 1.

The aim of the study was to evaluate promoter methylation of *CDKN1*, *CDKN2A*, *MYC*, *Smad3*, *SP1*, and *UBC* genes in tumor tissue of HNSCC patients.

## 2. Materials and Methods

### 2.1. Ethical Approval

All study participants were provided with patient-adapted information, and all patients signed an informed consent to participate in the study. Before patients were included in the study, the study protocol, patient information, and consent form were approved by an independent ethics committee (Extract from Minutes No. 634 of the Ethics Committee meeting of 17 November 2021. Extract from Minutes No. 684 of the Ethics Committee meeting of 2 March 2022) The study complies with the ethical standards developed in accordance with the World Medical Association Declaration of Helsinki “Ethical Principles for Scientific Medical Research Involving Human Subjects”, as amended in 2000, and the “Rules of Clinical Practice”. Participants were identified by patient number only.

### 2.2. Patient Selection

Fifty patients with HNSCC (37 men and 13 women) were included in this study. All patients underwent surgery as the first stage of therapy, during which biopsies of normal peritumoral tissue and tumor tissue were obtained. The main inclusion criterion was the absence of other treatments before surgery. Characterization of the patients is presented in Table 2. Squamous cell cancer of the tongue was observed in 14, in the larynx in 20, in the oral cavity in 6, in the floor of the mouth in 3, and in the maxillary sinus in 6 patients. 21 of 50 patients had a long history of smoking. All patients were HPV-negative. According to the patients and their relatives, the patients have never abused alcohol.

The mean age of men was 58 (32 ÷ 76) years, and of women, 60 (47 ÷ 79) years. The age distribution among all patients was as follows: 2 patients 30–39 years (4%), 4 patients 40–49 years (8%), 17 patients 50–59 years (34%), 18 patients 60–69 years (36%) and 7 patients 70–79 years (14%).

### 2.3. Sampling and DNA Extraction

Tumor and normal tissue samples from each patient were obtained during surgery and stored at −20 °C. DNA isolation from biomaterials was performed on microcolumns (K-SORB, № EX-514, Syntol, Moscow, Russia) according to the manufacturer’s instructions.

### 2.4. DNA Methylation Analysis

Bisulfite conversion was performed with the EZ DNA Methylation-Lightning kit (ThermoFisher EpiJET Bisulfite Conversion Kit, K1461, ThermoFisher Scientific, Waltham, MA, USA) according to the manufacturer’s instructions.

Methylation of the promoter regions of the genes was performed with the Methylation-Sensitive High-Resolution Melting (MS-HRM) method using the CFX 96 Connect Real-time System (BioRad, Hercules, CA, USA).

The primers for the reaction were selected using Primer Blast software (Table 3). The ready-mix (PCR-Mix, M-428, Syntol, Russia) was used for two-step PCR. Program of amplification was 95 °C—5 min; (95 °C—15 s, 60 °C—30 s, 72 °C—45 s) ×30 cycles; (95 °C—15 s, 50 °C—30 s, 72 °C—45 s) ×25 cycles. Further, the intercalating dye EVA Green (Syntol, Russia) was added to the obtained products. Each sample was run in duplicate. Construction of the melting curve was performed according to the following program: 1st stage—95°—30 s; 2nd stage—60°—10 min, 3rd stage—melting analysis in the range 60°–90° with 0.2° step. MS-HRM was performed using Precision Melt Analysis Software, version 3 (BioRad, USA). A CFX96 amplifier (BioRad, USA) was used for PCR and MS-HRM. The methylation level was detected by fluorescence expressed in relative fluorescence units (RFU).

### 2.5. Statistical Analysis

Statistical analysis of the data was carried out using R language (Version 4.2.3). The method used is the Chi-squared test. *p* values less than 0.05 were considered statistically significant.

## 3. Results

The results average of methylation level of the DNA promoter in CDKN1, CDKN2A, MYC, Smad3, SP1, and UBC genes is shown for all patient’s normal and tumor tissues and subgroups in Table 4. Significant differences in DNA methylation level between the patient’s tumor and normal tissues were found for the SP1 gene in all persons (*p* < 0.05).

We compared the average methylation levels for these genes in the following subgroups of patients (larynx, tongue, and other cancers, T2, T3, and T4, age before and after 50 years old, smokers, and non-smokers). Significant differences were observed for the *SP1* gene in different subgroups of the patient’s tumor and normal tissues (*p* < 0.05). For other genes, no significant differences were observed.

## 4. Discussion

Currently, hypermethylation of tumor suppressor gene promoters is the most characterized epigenetic event in carcinogenesis [11]. A number of studies have described hypermethylation of promoter regions of various genes in patients with HNSCC. The work of R. Noorlag et al. described a number of genes whose methylation changes contribute to the development of HNSCC [12].

Transcription factor specificity protein 1 (*Sp1*) regulates target genes by binding to the 5′-GGGGGCGG-3′ motif on their promoter [13]. This transcription factor was originally assigned an important role in regulating the transcription of a large number of “housekeeping genes”, so named because of their involvement in important cellular events: metabolism, cell proliferation/growth, and cell death [14]. It is estimated that the human genome contains, on average, more than 10,000 *Sp1* binding sites. In addition, *Sp1* is known to induce and inhibit transcription of a large number of genes [15]. *Sp1* activity is regulated throughout the cell cycle and is modulated by post-translational modifications in response to a variety of signals [16]. The protein encoded by this gene is involved in many cellular processes under both normal physiological conditions and pathology, including cell differentiation, cell growth, apoptosis, immune responses, DNA damage response, and chromatin remodeling. *Sp1* is overexpressed in cancer cells and, in most cases, activates genes that enhance proliferation, invasion, and chemoresistance [17]. *Sp1* is overexpressed in a number of cancers, including breast, gastric, pancreatic, lung, brain (glioma), and thyroid cancers [18,19,20,21]. In patient samples and cancer models, *Sp1* levels correlate with stage, invasive potential, and metastasis. *Sp1* levels correlate with patient survival in almost all cancers, with high *Sp1* levels associated with poor prognosis. In HNSCC, *Sp1* overexpression is also associated with tumor progression and is a negative prognostic factor. Thus, increased expression of this gene in HNSCC is associated with increased migration of cancer cells and invasive potential and, as a consequence, with rapid metastasis [22,23]. *Sp1* is involved in the regulation of HNSCC progression by controlling cell proliferation [24], apoptosis, cell migration, and invasion [25].

A large number of molecular genetic studies of HNSCC have revealed a number of differences between HPV-positive and HPV-negative samples [26]. HPV-positive HNSCC is characterized by more numerous alterations in gene expression profile or the appearance of somatic mutations in genes involved in cell survival and apoptosis, cell cycle, DNA replication, recombination and repair, nucleic acid metabolism, immune response, transcriptional and post-transcriptional regulation through the action of viral oncogenes or epigenetic silencing [27]. In turn, HPV-negative HNSCC is dominated by mutations that either inactivate tumor suppressor genes or enhance the function of oncogenes [28]. We hypothesize that the hypermethylation of this gene detected in this study is due to the fact that patients with HPV-negative HNSCC were included in the study.

The study of DNA methylation patterns in various genes involved in carcinogenesis is promising, as hypermethylated promoters may serve as potential biomarkers of disease. The Food and Drug Administration (FDA) has already approved a number of drugs for the treatment of haemablastosis targeting epigenetic alterations. These drugs are mainly DNA methylation inhibitors, such as vidase and dacogen (Decitabine) and others [29,30]. A number of works are devoted to describing the efficacy of drugs targeting demethylation in solid tumors [31,32,33]. In addition to this, studies aimed at determining the prognostic significance of methylation of certain genes in response to drug antitumor therapy are actively conducted [34,35,36,37].

DNA methylation profiling can serve as a new tool in oncology to improve the classification of HNSCC and predict response to existing treatment strategies, as well as to identify targets for the creation of new targeted drugs [38].

## Figures and Tables

**Table 1:** Characteristic of studied genes.

Gene Name	Synonyms:	Location	MIM	Exon Count	Gene ID	Transcripts	Gene Type	Gene Function
*CDKN1A*–cyclin dependent kinase inhibitor 1A	*CAP20*, *CDKN1*, *CIP1*, *MDA-6*, *P21*, *SDI1*, *WAF1*, *p21CIP1*	6p21.2	116899	6	1026	0 REFSEQ mRNAs: NM_000389.5 NM_001220777.2 NM_001220778.2 NM_001291549.3 NM_001374509.1	Protein coding	Inhibition of cellular proliferation, cyclin-dependent kinase activity, DNA synthesis by DNA polymerase delta; Blocking and controlling cell cycle.
*CDKN2A* cyclin dependent kinase inhibitor 2A	*ARF, CAI2, CDK4I, CDKN2, CMM2, INK4, INK4A, MLM, MTS-1, MTS1, P14, P14ARF, P16, P16-INK4A, P16INK4, P16INK4A, P19, P19ARF, TP16*	9p21.3	600160	10	1029	NM_000077.5 NM_001195132.2 NM_001363763.2 NM_058195.4 NM_058196.1	Protein coding, tumor suppressor	Cell cycle arrest in G1 and G2 phases; controlling cell proliferation and apoptosis; inhibiting ribosome biogenesis.
*MYC* MYC proto-oncogene, bHLH transcription factor	*MRTL, MYCC, bHLHe39, c-Myc*	8q24.21	190080	3	4609	2 REFSEQ mRNAs: NM_001354870.1 NM_002467.6	Protein coding	Transcription activation of growth-related genes; regulation of somatic reprogramming, controlling self-renewal of embryonic stem cells.
*SMAD3* SMAD family member 3	*LDS3; mad3; LDS1C; MADH3; JV15-2; hMAD-3; hSMAD3; HSPC193; HsT17436*	15q22.33	603109	15	4088	11 REFSEQ mRNAs: NM_001145102.2 NM_001145103.2 NM_001145104.2 NM_001407011.1 NM_001407012.1 NM_001407013.1 NM_001407014.1 NM_001407015.1 NM_001407016.1 NM_001407017.1 NM_005902.4	Protein coding	Regulation of chondrogenesis and osteogenesis; binding the TRE element in the promoter region of many genes; Positive regulation PDPK1 kinase activity
*SP1* *Sp1* transcription factor	no	12q13.13	189906	7	6667	3 REFSEQ mRNAs: NM_001251825.2 NM_003109.1 NM_138473.3	Protein coding	Regulation the expression, binding with high affinity to GC-rich motifs; modulating the cellular response to DNA damage; chromatin remodeling; protecting cells against oxidative stress.
*UBC* ubiquitin C	*HMG20*	12q24.31	191340	2	7316	1 REFSEQ mRNAs: NM_021009.7	Protein coding	DNA replication; Protein ubiquitination; post-translational protein modification; transcription-coupled nucleotide excision repair (TC-NER)

**Table 2:** Characteristics of patients (n = 50). M—male, F—female.

Patient ID	Tumor Origin	ICD-10	TNM Classification			Gender	Age	Smoker
			T	N	M			
1	Tongue n = 16	C02.0	1	0	0	M	50	yes
2		C02.0	3	0	0	F	53	yes
3		C02.1	2	0	0	F	59	no
4		C02.1	2	0	0	M	71	yes
5		C02.1	3	0	0	F	47	no
6		C02.1	3	0	0	M	63	no
7		C02.1	3	0	0	M	40	no
8		C02.1	3	0	0	M	46	no
9		C02.1	3	0	0	M	47	yes
10		C02.1	3	1	0	M	68	no
11		C02.1	3	2b	0	F	60	No
12		C02.1	3	0	0	F	69	Yes
13		C02.1	3	0	0	M	60	No
14		C02.1	3	0	0	F	79	No
15		C02.1	3	0	0	M	64	Yes
16		C02.1	4	2	0	M	36	No
17	Larynx n = 21	C32.0	2	0	0	F	55	No
18		C32.0	3	0	0	M	69	No
19		C32.0	3	0	0	M	59	Yes
20		C32.0	3	0	0	M	62	No
21		C32.0	3	1	0	M	49	No
22		C32.0	3	0	0	M	70	No
23		C32.0	3	1	0	M	58	Yes
24		C32.0	4a	0	0	M	71	Yes
25		C32.0	4a	2б	0	M	64	No
26		C32.0	4a	2c	0	M	59	Yes
27		C32.0	4a	1	0	M	60	No
28		C32.1	3	2b	0	M	64	Yes
29		C32.1	4a	2б	0	M	76	Yes
30		C32.8	2	0	0	M	50	Yes
31		C32.8	3	0	0	M	58	Yes
32		C32.8	3	0	0	M	50	No
33		C32.8	3	1	0	M	58	No
34		C32.8	3	1	0	M	59	No
35		C32.8	3	0	0	M	59	No
36		C32.8	4	0	0	M	72	Yes
37	Gum n = 3	C03.0	2	0	0	F	53	No
38		C03.1	3	0	0	M	66	No
39		C03.1	4a	0	0	M	50	Yes
40	Floor of mouth n = 5	C04.1	2	0	0	F	64	Yes
41		C04.1	2	0	0	F	64	No
42		C04.1	3	0	0	M	60	Yes
43		C04.1	3	0	0	F	66	No
44		C04.8	4a	1	0	M	32	No
45	Cheek mucosa n = 3	C06.0	2	0	0	M	63	Yes
46		C06.0	3	1	0	M	74	Yes
47		C06.2	2	0	0	F	55	No
48	Maxillary sinus n = 3	C31.0	4	0	0	M	40	No
49		C31.0	3	1	0	F	60	No
50		C31.0	4a	1	0	M	53	Yes

**Table 3:** Characteristics of primers used.

Gene	Forward Primer Sequence (5′ → 3′)	Reverse Primer Sequence (5′ → 3′)	Product Size (bp)
*CDKN1A*	ATTAGTTGGGTATGGTGGTGTATGT	ACCCAAACATATTCCTAAAAAACAA	540
*CDKN2A*	TTTTTAGTTGGAAAGGAGGAAGG	TCCTCTTCTAAATTTAAAAAACAAAC	573
*MYC*	TTAATAATAAAAGGGGAAAGAGGATTT	CAAACTAAATCCCCCAATTTACTAC	516
*Smad3*	GTTTAAGGGGAAGAAGAGAAAGAGT	AACTACACCCAACTACCTAAATCAC	550
*SP1*	TTATTGGTTTTTAATATTGAGAGGG	AACTTAAAATAAACTCATCCTTACC	363
*UBC*	TTTTTAGATAGTTTTATGGGGTTGG	ACTCAAAAATCAAATATCAAATCAC	412

**Table 4:** The average level of promotor gene methylation in tumor and normal tissue in all patient and their subgroups. T-tumor, N-normal.

Genes	Sample	All (n = 50)	TNM Classification **			Tumor Origin			Age		Smokers	
									Under 50 Years (n = 12)	Over 50 Years (n = 38)	Yes	No
			T_2_ (n = 9)	T_3_ (n = 29)	T_4_ (n = 11)	Tongue (n = 16)	Larynx (n = 20)	Other (n = 14)			(n = 21)	(n = 29)
		M ± m, Range										
*CDKN1A*	T	0.35 ± 0.21 (0.01 ÷ 1.0)	0.37 ± 0.21 (0.04 ÷ 0.6)	0.36 ± 0.21 (0.01 ÷ 0.62)	0.31 ± 0.19 (0.01 ÷ 1.0)	0.37 ± 0.17 (0.01 ÷ 0.56)	0.35 ± 0.17 (0.04 ÷ 0.57)	0.33 ± 0.26 (0.01 ÷ 1.0)	0.33 ± 0.19 (0.04 ÷ 0.58)	0.35 ± 0.22 (0.01 ÷ 1.0)	0.36 ± 0.24 (0.01 ÷ 0.62)	0.34 ± 0.19 (0.01 ÷ 1.0)
	N	0.29 ± 0.19 (0.01 ÷ 0.67)	0.33 ± 0.19 (0.06 ÷ 0.57)	0.28 ± 0.20 (0.04 ÷ 0.67)	0.25 ± 0.18 (0.01 ÷ 0.52)	0.24 ± 0.20 (0.01 ÷ 0.55)	0.26 ± 0.20 (0.06 ÷ 0.64)	0.35 ± 0.19 (0.01 ÷ 0.67)	0.28 ± 0.20 (0.01 ÷ 0.67)	0.28 ± 0.20 (0.01 ÷ 0.64)	0.27 ± 0.18 (0.01 ÷ 0.67)	0.27 ± 0.22 (0.01 ÷ 0.64)
*CDKN2A*	T	0.28 ± 0.09 (0.06 ÷ 0.50)	0.23 ± 0.09 (0.06 ÷ 0.36)	0.30 ± 0.09 (0.13 ÷ 0.50)	0.29 ± 0.09 (0.14 ÷ 0.41)	0.28 ± 0.09 (0.14 ÷ 0.49)	0.30 ± 0.09 (0.13 ÷ 0.47)	0.27 ± 0.09 (0.06 ÷ 0.50)	0.30 ± 0.08 (0.14 ÷ 0.42)	0.28 ± 0.09 (0.06 ÷ 0.50)	0.27 ± 0.05 (0.06 ÷ 0.50)	0.31 ± 0.10 (0.14 ÷ 0.36)
	N	0.25 ± 0.13 (0.01 ÷ 0.59)	0.22 ± 0.13 (0.01 ÷ 0.44)	0.26 ± 0.13 (0.10 ÷ 0.59)	0.26 ± 0.12 (0.12 ÷ 0.56)	0.28 ± 0.14 (0.10 ÷ 0.59)	0.22 ± 0.13 (0.01 ÷ 0.56)	0.28 ± 0.10 (0.10 ÷ 0.46)	0.28 ± 0.14 (0.12 ÷ 0.56)	0.25 ± 0.12 (0.01 ÷ 0.59)	0.25 ± 0.12 (0.01 ÷ 0.56)	0.26 ± 0.13 (0.10 ÷ 0.59)
*MYC*	T	0.12 ± 0.05 (0.01 ÷ 0.27)	0.13 ± 0.05 (0.07 ÷ 0.24)	0.12 ± 0.05 (0.01 ÷ 0.27)	0.13 ± 0.05 (0.08 ÷ 0.17)	0.12 ± 0.05 (0.06 ÷ 0.26)	0.12 ± 0.04 (0.07 ÷ 0.24)	0.13 ± 0.06 (0.01 ÷ 0.27)	0.11 ± 0.04 (0.03 ÷ 0.17)	0.12 ± 0.05 (0.01 ÷ 0.27)	0.11 ± 0.05 (0.03 ÷ 0.28)	0.13 ± 0.05 (0.01 ÷ 0.24)
	N	0.12 ± 0.05 (0.04 ÷ 0.33)	0.10 ± 0.05 (0.04 ÷ 0.18)	0.12 ± 0.05 (0.05 ÷ 0.21)	0.12 ± 0.04 (0.06 ÷ 0.33)	0.10 ± 0.03 (0.04 ÷ 0.20)	0.13 ± 0.03 (0.06 ÷ 0.19)	0.12 ± 0.06 (0.06 ÷ 0.33)	0.12 ± 0.04 (0.06 ÷ 0.21)	0.12 ± 0.05 (0.04 ÷ 0.33)	0.11 ± 0.02 (0.04 ÷ 0.33)	0.12 ± 0.06 (0.07 ÷ 0.16)
*Smad3*	T	0.69 ± 0.19 (0.01 ÷ 0.83)	0.68 ± 0.16 (0.01 ÷ 0.83)	0.68 ± 0.18 (0.01 ÷ 0.83)	0.72 ± 0.25 (0.61 ÷ 0.81)	0.69 ± 0.19 (0.01 ÷ 0.83)	0.72 ± 0.27 (0.01 ÷ 0.83)	0.65 ± 0.008 (0.04 ÷ 0.79)	0.74 ± 0.05 (0.61 ÷ 0.81)	0.68 ± 0.21 (0.01 ÷ 0.83)	0.67 ± 0.17 (0.01 ÷ 0.83)	0.71 ± 0.19 (0.01 ÷ 0.79)
	N	0.65 ± 0.17 (0.32 ÷ 0.82)	0.64 ± 0.17 (0.32 ÷ 0.81)	0.65 ± 0.16 (0.32 ÷ 0.82)	0.63 ± 0.19 (0.32 ÷ 0.80)	0.65 ± 0.17 (0.32 ÷ 0.81)	0.62 ± 0.11 (0.38 ÷ 0.81)	0.71 ± 0.18 (0.32 ÷ 0.82)	0.67 ± 0.17 (0.32 ÷ 0.80)	0.65 ± 0.16 (0.32 ÷ 0.82)	0.64 ± 0.16 (0.32 ÷ 0.82)	0.67 ± 0.16 (0.32 ÷ 0.79)
*SP1*	T	0.22 ± 0.10 * (0.09 ÷ 0.45)	0.22 ± 0.10 * (0.01 ÷ 0.38)	0.21 ± 0.11 * (0.01 ÷ 0.42)	0.23 ± 0.09 * (0.01 ÷ 0.45)	0.20 ± 0.09 * (0.11 ÷ 0.42)	0.24 ± 0.08 * (0.11 ÷ 0.45)	0.21 ± 0.11 * (0.09 ÷ 0.42)	0.23 ± 0.12 * (0.12 ÷ 0.45)	0.21 ± 0.09 * (0.09 ÷ 0.42)	0.22 ± 0.10 * (0.11 ÷ 0.45)	0.21 ± 0.10 * (0.09 ÷ 0.42)
	N	0.11 ± 0.06 (0.01 ÷ 0.23)	0.11 ± 0.09 (0.01 ÷ 0.27)	0.11 ± 0.09 (0.04 ÷ 0.42)	0.09 ± 0.06 (0.01 ÷ 0.30)	0.10 ± 0.05 (0.04 ÷ 0.20)	0.09 ± 0.05 (0.01 ÷ 0.23)	0.13 ± 0.05 (0.01 ÷ 0.17)	0.11 ± 0.05 (0.04 ÷ 0.20)	0.11 ± 0.05 (0.01 ÷ 0.23)	0.11 ± 0.05 (0.01 ÷ 0.19)	0.10 ± 0.05 (0.01 ÷ 0.23)
*UBC*	T	0.34 ± 0.23 (0.01 ÷ 0.72)	0.36 ± 0.23 (0.18 ÷ 0.52)	0.33 ± 0.23 (0.01 ÷ 0.72)	0.36 ± 0.11 (0.01 ÷ 0.72)	0.28 ± 0.23 (0.01 ÷ 0.72)	0.34 ± 0.21 (0.01 ÷ 0.72)	0.41 ± 0.24 (0.01 ÷ 0.72)	0.35 ± 0.23 (0.01 ÷ 0.72)	0.34 ± 0.23 (0.01 ÷ 0.72)	0.37 ± 0.23 (0.01 ÷ 0.72)	0.32 ± 0.22 (0.01 ÷ 0.72)
	N	0.16 ± 0.08 (0.01 ÷ 0.75)	0.43 ± 0.25 (0.02 ÷ 0.75)	0.27 ± 0.25 (0.01 ÷ 0.72)	0.26 ± 0.25 (0.01 ÷ 0.65)	0.27 ± 0.26 (0.01 ÷ 0.70)	0.24 ± 0.23 (0.01 ÷ 0.75)	0.39 ± 0.25 (0.01 ÷ 0.72)	0.23 ± 0.21 (0.02 ÷ 0.58)	0.31 ± 0.26 (0.01 ÷ 0.75)	0.26 ± 0.27 (0.01 ÷ 0.72)	0.31 ± 0.24 (0.01 ÷ 0.75)

* Significant differences between tumor and normal tissues (*p* < 0.05). M—mean, m—standard deviation. ** One patient had T1N0M0 stage with average methylation levels in CDKN1, CDKN2A, MYC, Smad3, SP1, and UBC genes in the patient’s tumor tissue equal to 0.26, 0.32, 0.08, 0.78, 0.42, 0.01 respectively, and in patient’s normal tissue equal to 0.26, 0.22, 0.12, 0.74, 0.10 and 0.03 respectively.

## Data Availability

The findings have not been published earlier.
